# Publisher Correction: Mutant KRAS vaccine with dual checkpoint blockade in resected pancreatic cancer: a phase I trial

**DOI:** 10.1038/s41467-026-71968-x

**Published:** 2026-04-15

**Authors:** Amanda L. Huff, S. Daniel Haldar, Alexander A. Girgis, Hejia Henry Wang, Ludmila Danilova, Thatcher Heumann, Maureen Berg, Yuxuan Wang, Lalitya Andaloori, Alexei Hernandez, Gabriella Longway, Benjamin Barrett, Zirui Zhu, Emily Davis-Marcisak, Christopher Thoburn, James Leatherman, Sarah Mitchell, Jae W. Lee, Daniel H. Shu, Maximillian F. Konig, Brian J. Mog, Janelle Montagne, Erin M. Coyne, Katherine Bever, Marina Baretti, Mark Yarchoan, Robert A. Anders, Luciane T. Kagohara, Daniel Laheru, Amy M. Thomas, Jennifer Durham, Julie M. Nauroth, Jiayun Lu, Hao Wang, Elana J. Fertig, Won Jin Ho, Nilofer S. Azad, Elizabeth M. Jaffee, Neeha Zaidi

**Affiliations:** 1 Johns Hopkins Convergence Institute, Johns Hopkins University School of Medicine, Baltimore, MD USA; 2 Johns Hopkins Bloomberg Kimmel Institute for Immunotherapy, Johns Hopkins University School of Medicine, Baltimore, MD USA; 3 Department of Oncology, Sidney Kimmel Comprehensive Cancer Center, Johns Hopkins University, Baltimore, MD USA; 4 Department of Gastrointestinal Medical Oncology, The University of Texas MD Anderson Cancer Center, Houston, TX USA; 5 Department of Genetic Medicine, Johns Hopkins University School of Medicine, Baltimore, MD USA; 6 Department of Pathology, Johns Hopkins University School of Medicine, Baltimore, MD USA; 7 Greenebaum Comprehensive Cancer Center, University of Maryland School of Medicine, Baltimore, MD USA; 8 Ludwig Center, Sidney Kimmel Comprehensive Cancer Center, Johns Hopkins University School of Medicine, Baltimore, MD USA; 9 Lustgarten Pancreatic Cancer Research Laboratory, Sidney Kimmel Comprehensive Cancer Center, Johns Hopkins University School of Medicine, Baltimore, MD USA; 10 Howard Hughes Medical Institute, Chevy Chase, MD USA; 11 Division of Rheumatology, Department of Medicine, Johns Hopkins University School of Medicine, Baltimore, MD USA

**Keywords:** Pancreatic cancer, Tumour vaccines, Drug development, Cancer therapy

Correction to: *Nature Communications* 10.1038/s41467-026-68324-4, published online 10 February 2026

In this article, the author’s name Alexander A. Girgis was incorrectly written as Alexander A. Gergis. The original article has been corrected.

