# Supplementary material for: Mutant KRAS vaccine with dual checkpoint blockade in resected pancreatic cancer: a phase I trial
Source: Nat Commun. 2026 Feb 10;17:1538. doi: 10.1038/s41467-026-68324-4 (PMC12891733; doi:10.1038/s41467-026-68324-4)
Supplement: Supplementary file 1 — Supplementary Information [file 41467_2026_68324_MOESM1_ESM.pdf]

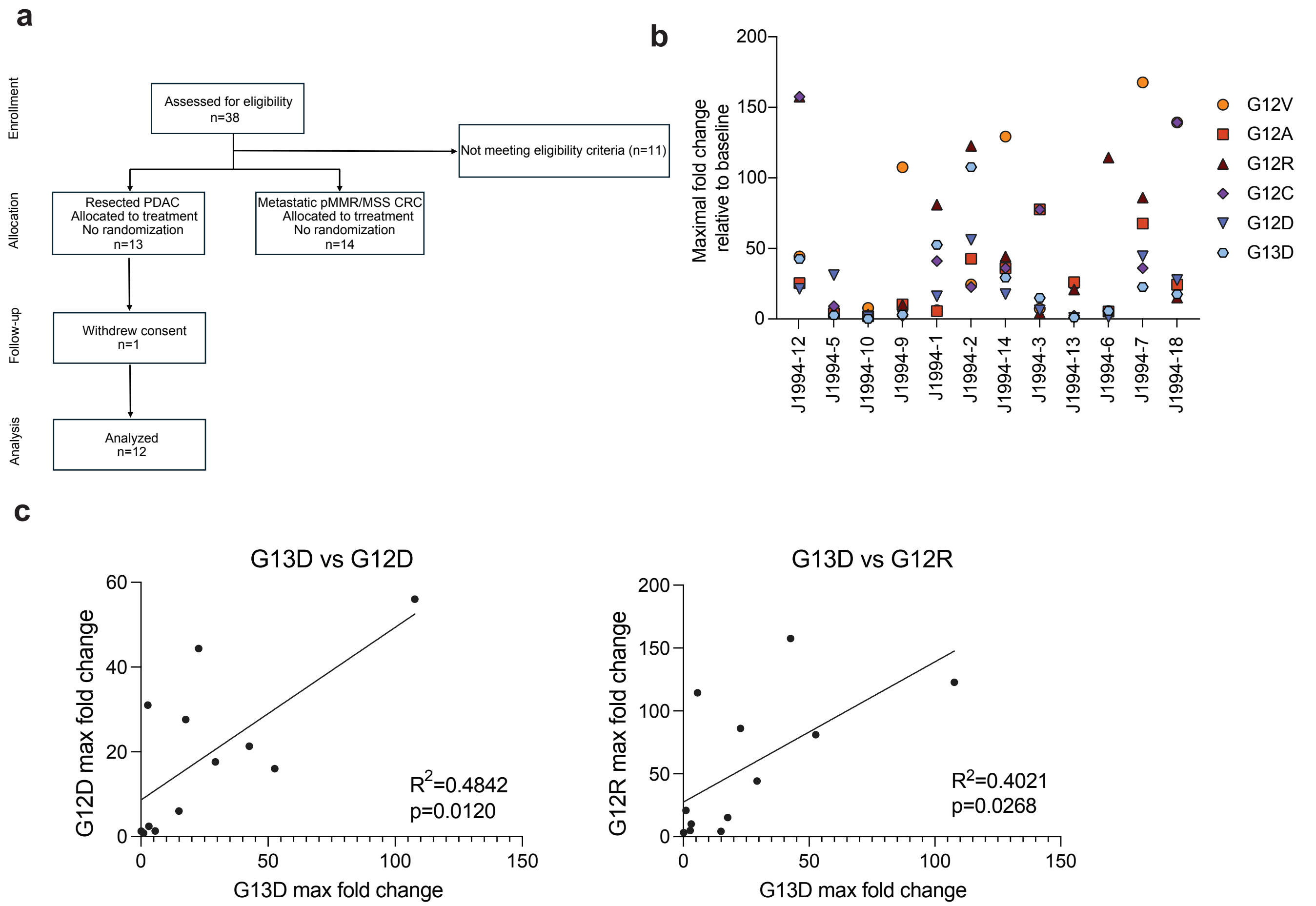

Supplementary Figure 1- Patient disposition and maximal fold change responses to mKRAS-VAX. a) Patient screening, enrollment, and treatment overview. **b)** Maximal fold change relative to baseline for each mKRAS mutation for each patient. For each patient, mutant KRAS specific T cell response data was normalized by subtracting the corresponding number of IFNγ SFU detected in the control peptide from each mKRAS peptide stimulation, then adding 1. When data was <1.0, it was replaced with 1.0. The absolute change in IFNγ SFU in each mKRAS peptide stimulation was then normalized to the absolute change at baseline sample by division to determine fold change. **c)** Correlation of maximal fold change response for KRAS G13D relative to KRAS G12D (left) or G12R (right) for each patient.

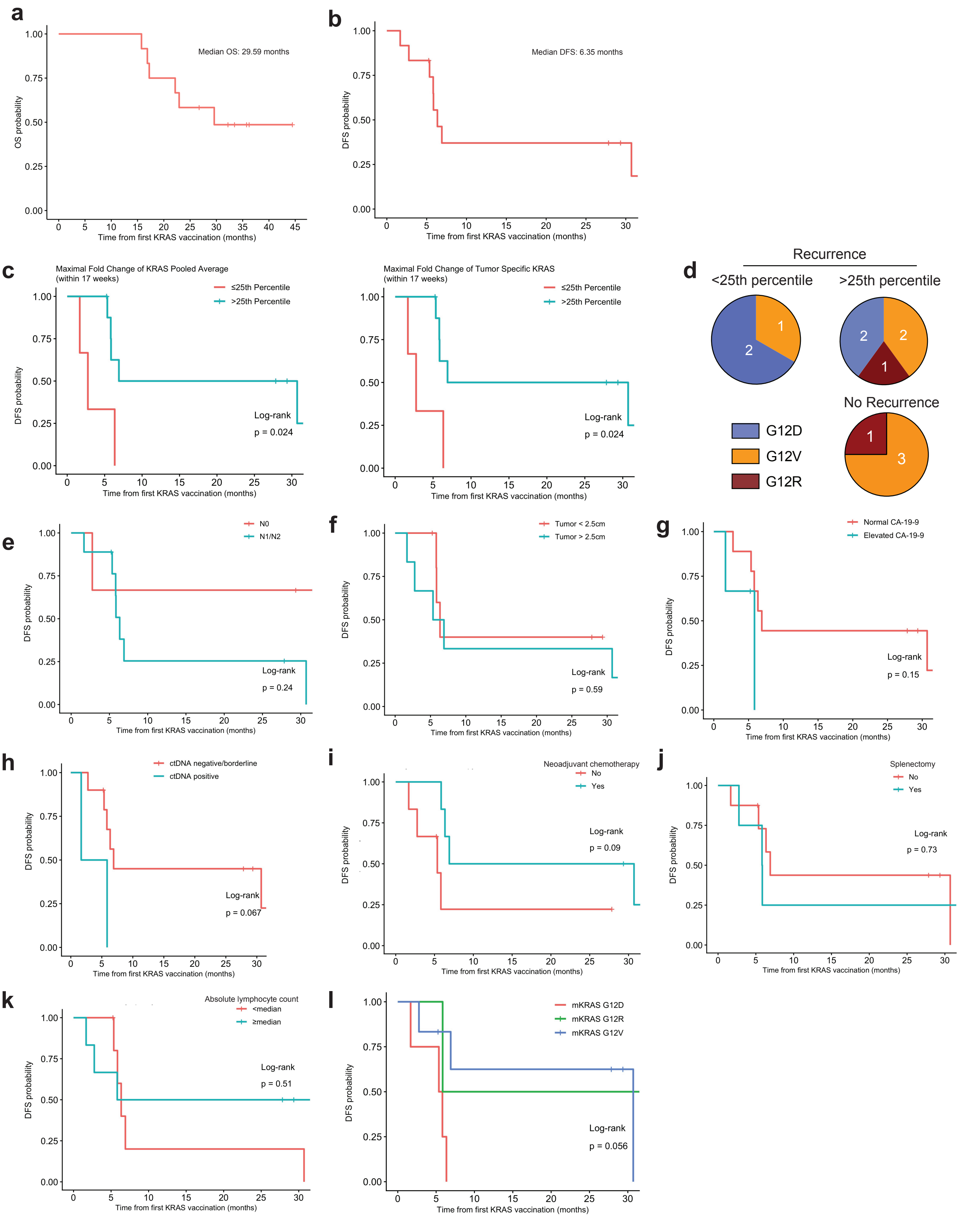

Supplementary Figure 2- Clinical outcomes of patients vaccinated with mKRAS-VAX. a) Overall survival (OS) and **b)** disease free survival (DFS) for all patients (n=12). Time of data cut off was 10/24/2024. **c)** Disease-free survival (DFS) of patients who were determined to be in the lower quartile (red) or upper three quartiles (blue) of average mKRAS response (left) or patient mutation-specific response. **d)** Distribution of the tumor mutation type for patients who recurred (n=8) while on trial including those who were within the lower quartile (n=3) or upper three quartiles (n=5) of tumor mutation-specific IFNγ response or for patients who did not recur (n=4).Correlation of DFS with **e)** lymph node at time of resection **f)** size of primary tumor, **g)** baseline CA19-9 levels, **h)** minimal residual disease status measured by ctDNA positivity in plasma at baseline, **i)** neoadjuvant chemotherapy status, **j)** splenectomy status, **k)** absolute lymphocyte count, or **l)** KRAS mutation status. Signficance was calculated using log-ranked test, p values are shown.

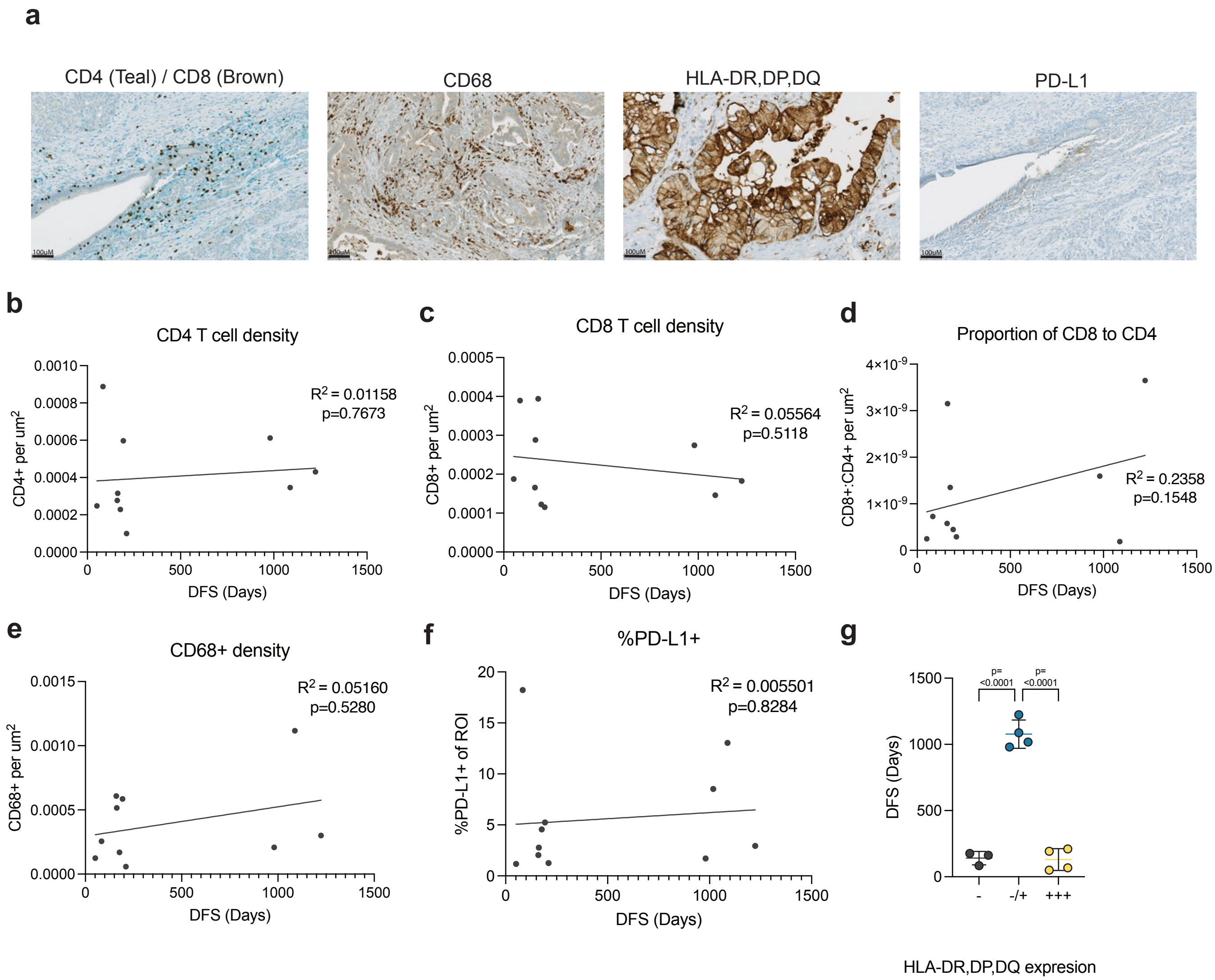

Supplementary Figure 3- Baseline immunological features of primary resected tumors. a) Representative images of IHC staining of primary tumor specimens costained for anti-CD4 and anti-CD8, anti-CD68 single stain, anti-HLA-DR,DP,DQ single stain, or anti-PD-L1 single stain. Scale bar represents 100uM. Density of marker staining was correlated with DFS reported in days for **b)** CD4 T cell density (n=10 patients), **c)** CD8 T cell density (n=10 patients), **d)** proportion of CD8 to CD4 T cell density (n=10 patients), **e)** macro-phage density (CD68+, n=10 patients), or **f)** %PD-L1+ in the tumor (n=11 patients). Linear regression models were performed. R squared value and p values are shown for each fit curve. **g)** Comparison of DFS with level of HLA-DR,DP,DQ staining within the tumor (n=11 patients). Patients were categorized into low/negative, moderate, or high expression of HLA-DR,DP,DQ within the tumor two independent expert pathologists. One way ANOVA was performed followed by Tukey's multiple comparisons.

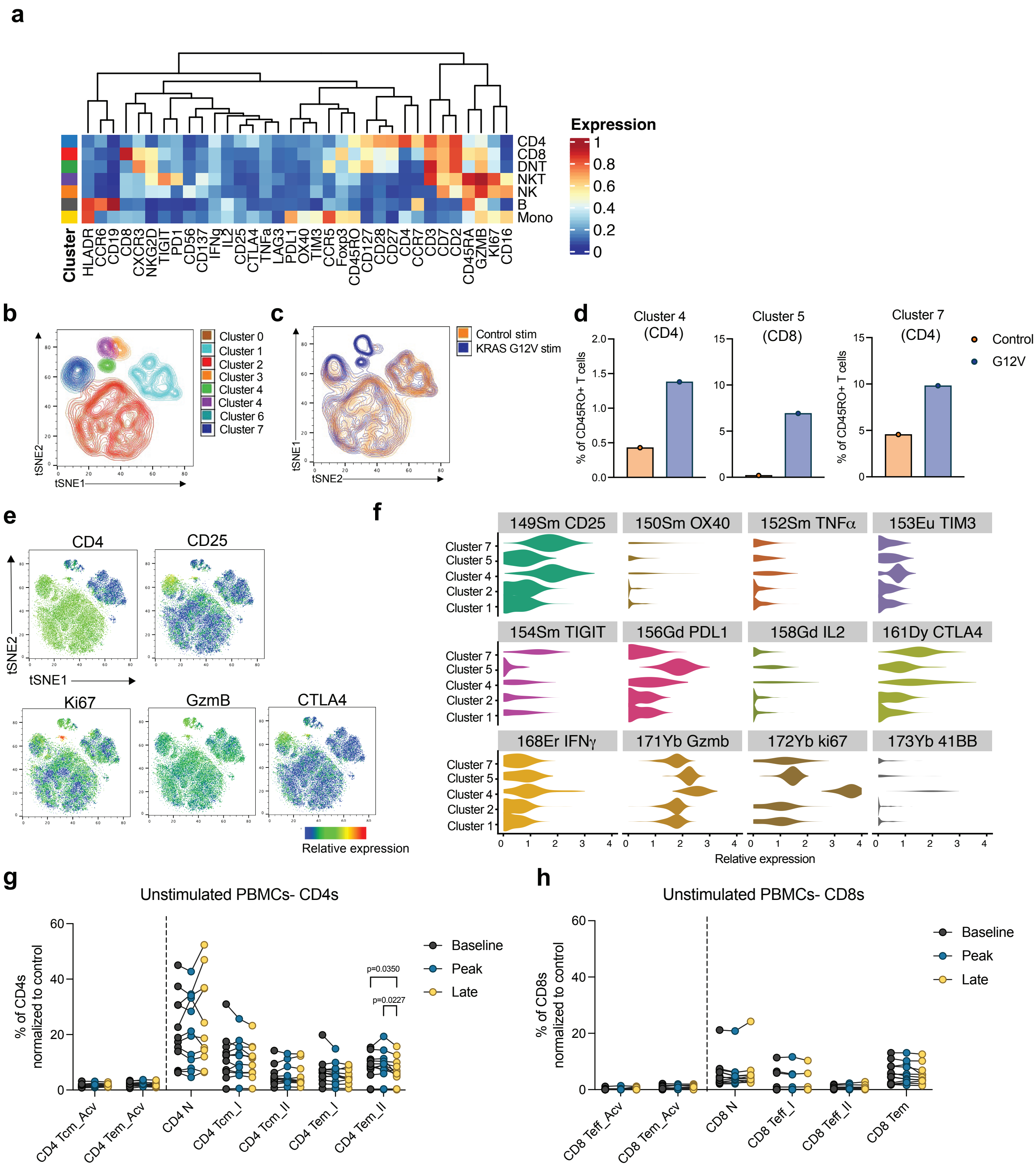

Supplementary Figure 4- CyTOF phenotyping of peptide restimulated T cells and functional marker expression correlation with recurrence. a) General cell type analysis of CyTOF restimulated PBMCs using unsupervised clustering of all samples. Cell types identified include Monocytes (Mono, CD16+ HLA-DR+), B cells (B, CD19+ HLA-DR+), Natural Killer (NK, CD16+), Natural Killer T cells (CD16+ CD3+), double negative T cell (DNT, CD3+CD4-CD8-), CD8 T cell (CD3+CD8+) or CD4 T cells (CD3+CD4+). **b)** Subset analysis of patient J1994_12 PBMCs four weeks post vaccination restimulated with control peptide or KRAS G12V. tSNE1 plots subset on CD3+ antigen experienced CD45RO+ T cells distinguishing 7 unique clusters. **c)** tSNE plot of unique T cell clusters distinguished by antigen stimulation condition. **d)** Quantification of T cell clusters with increased population after peptide restimulation relative to control peptide. **e)** tSNE1 plots with heatmap overlay expression of CD4, CD25, Ki67, Granzyme B, or CTLA4 for T cell clusters identified. **f)** Violin plot expression of activation marker, cytokine marker, proliferation, and exhaustion marker expression on a subset of T cell clusters. **g)** Percent of CD4 or **h)** CD8 T cell populations identified by unsupervised clustering methods in Figure 3 in unstimulated PBMCs for all patients (n=12). Mixed effects analysis followed by Tukey's multiple comparisons was performed.

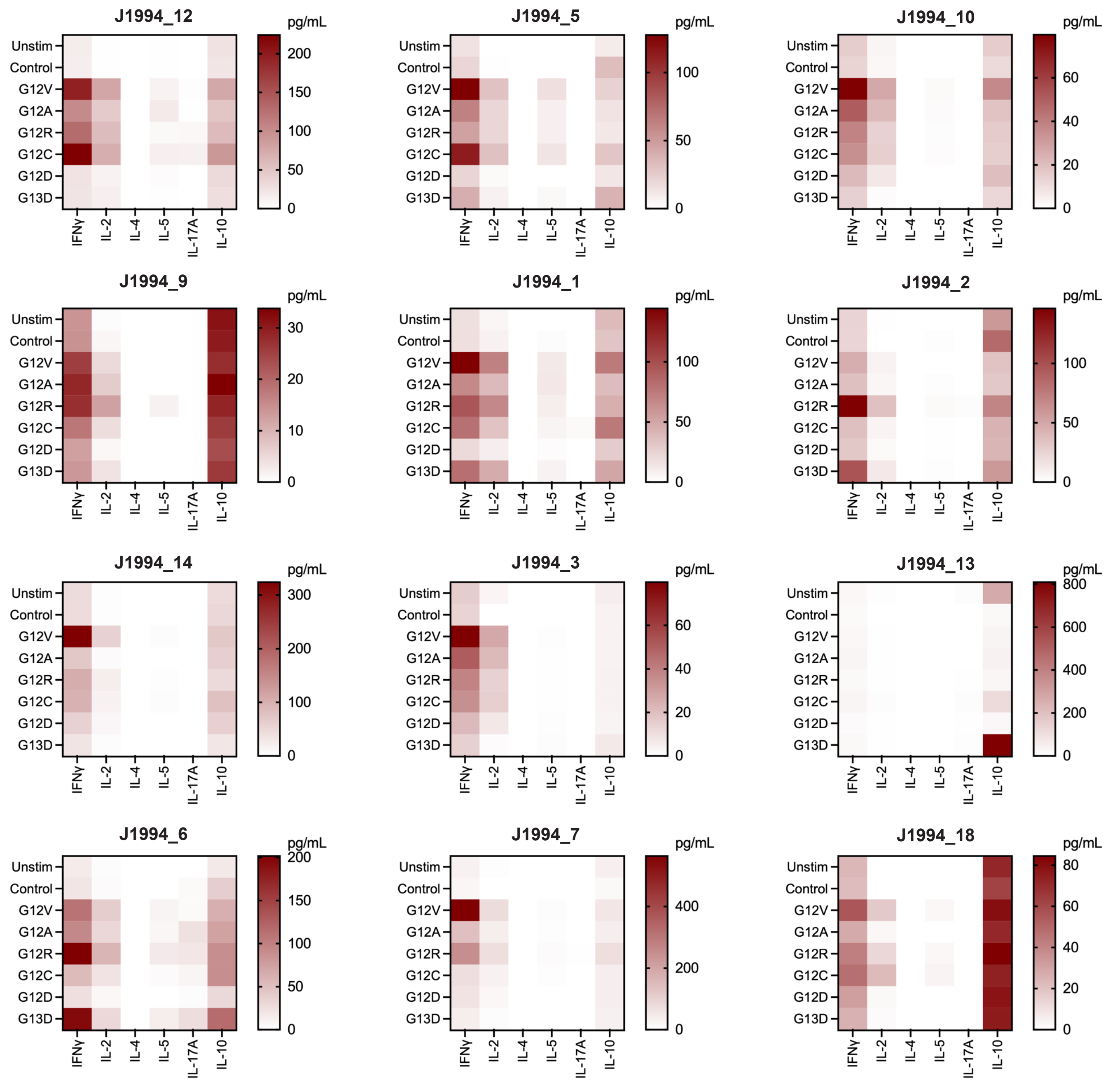

Supplementary Figure 5- Cytokines associated with a Th1 phenotype response are the predominant cytokines associated with mKRAS-specific T cell activation. Supernatants from unstimulated (no peptide), peptide restimulation and CyTOF assay performed in Figure 3 were analyzed by cytokine secretion for IFNγ, IL-2, IL-4, IL-5, IL-17A, and IL-10. Samples were ran as technical duplicates.

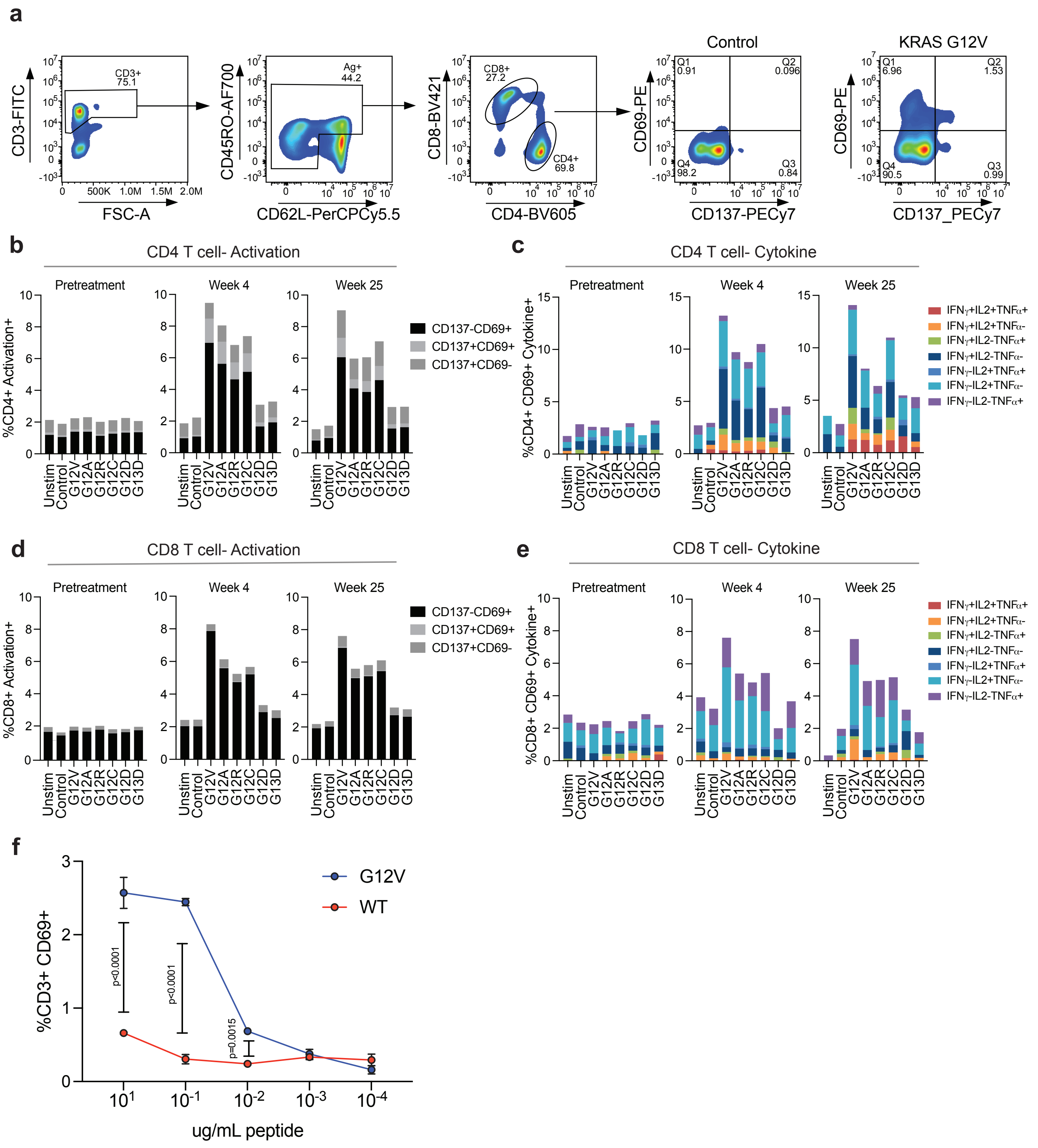

Supplementary Figure 6- Validation of responding CD4 and CD8 T cell populations by flow cytometry. a) Example of populations gated on by T cells (CD3+), Antigen experienced (CD45RO+CD62L+), and activation marker (CD69+, CD137+, CD4 T cell activation shown) expression used to evaluate KRAS-peptide responsive T cells in patient PBLs collected prior to vaccination, week 4, and week 25 post vaccination. PBLs were stimulated with 2ug/mL of individual KRAS peptides for 48 hours prior to flow staining. **b)** CD4 T cell activation marker (CD69, CD137) and **c)** Cytokine expression (IFNγ, IL2, TNFα) after 48h restimulation with 2ug/mL of patient PBLs with individual mKRAS SLPs. **d)** CD8 T cell activation marker (CD69, CD137) or **e)** cytokine expression (IFNγ, IL2, TNFα) after 48h restimulation with 2ug/mL of patient PBLs with individual mKRAS SLPs. **f)** Titration of peptide restimulation post-vaccine PBMCs with KRAS G12V or KRAS WT peptide at decreasing concentrations of peptide. CD3+ events were analyzed for CD69+ expression 48h after restimulation.

!!

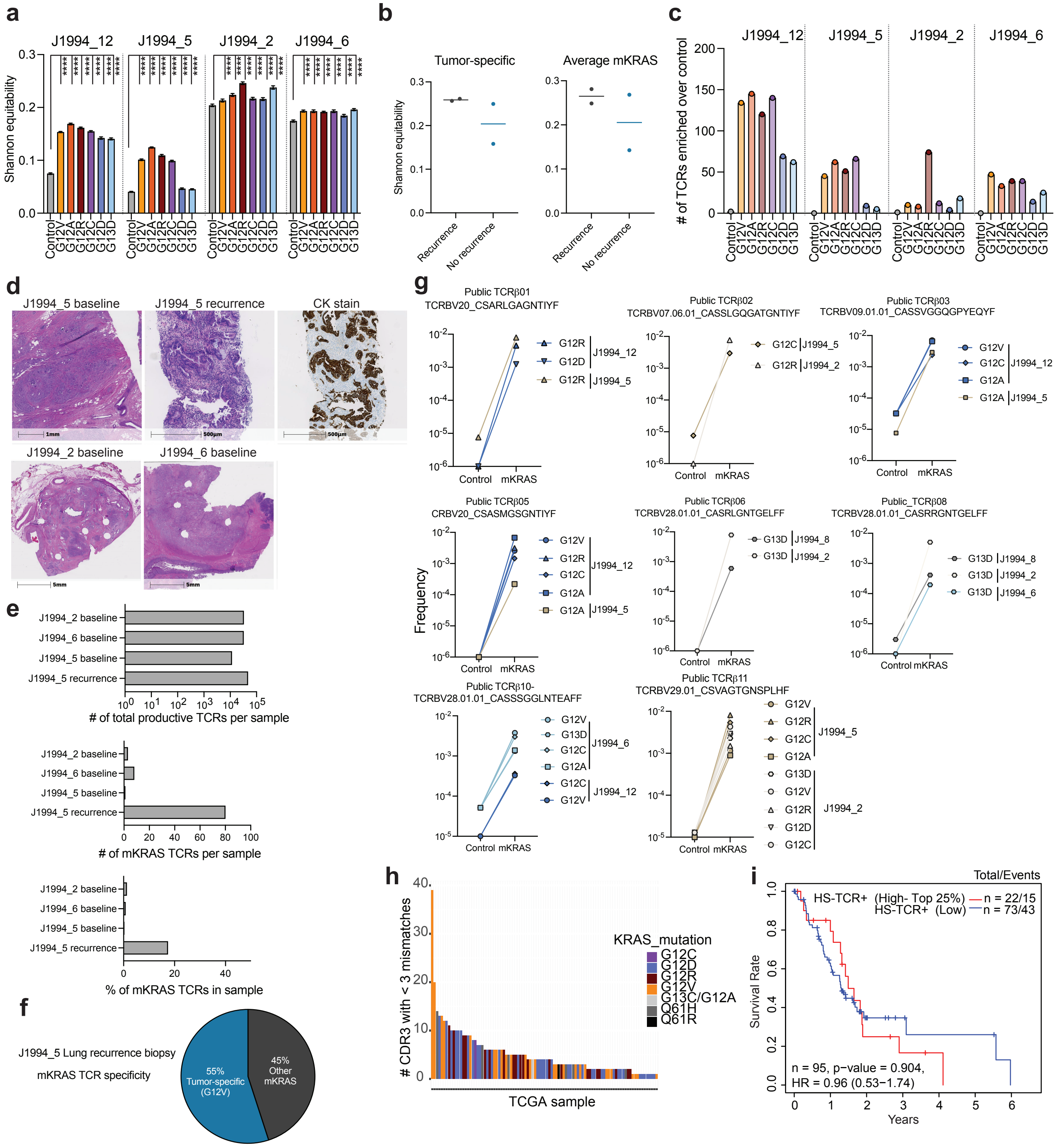

Supplementary Figure 7- mKRAS-specific TCRβ repertoire identified by in vitro peptide expansion. a) Shannon equitability of TCRβ chain repertoire in the control peptide expansion condition or each individual mKRAS peptide expansion for four patients. **b)** Shannon equitability of mKRAS-specific expanded TCRβ sequences for patients who recurred or did not recurr while on trial. **c)** Number of TCRs identified as significantly enriched relative to a hIL-2 only control in the control peptide expansion or for each mKRAS peptide for four patients. **d)** H&E of primary tumor sections or lung metastisis biopsy for patients with TCRβ sequencing **e)** Total productive TCR sequences or mKRAS-specific TCRs found in each tissue sample. **f)** Fraction of tumor-specific (G12V-reactive) TCRs found in J1994_5 lung recurrence biopsy relative to all of mKRAS-reactivities **g)** Frequency of the public TCRβ sequences identified in the no peptide control condition or mKRAS expansion for more than one patient. mKRAS antigen expansion is indicated by icon shape and patient it was identified in is indicated by color. **h)** Number of high similarity TCRβ CDR3 sequences (2 or fewer amino acid differences) identified from the mKRAS-peptide expansion assay in PDAC mKRAS positive TCGA tumor samples. TCGA samples are colored by tumor mutation present in tumor. **i)** Log-rank analysis of overall survival of tumor for patients with mKRAS+ tumors in the TCGA PAAD dataset stratified into high (Top 25%) or low abundance of HS-TCRs.

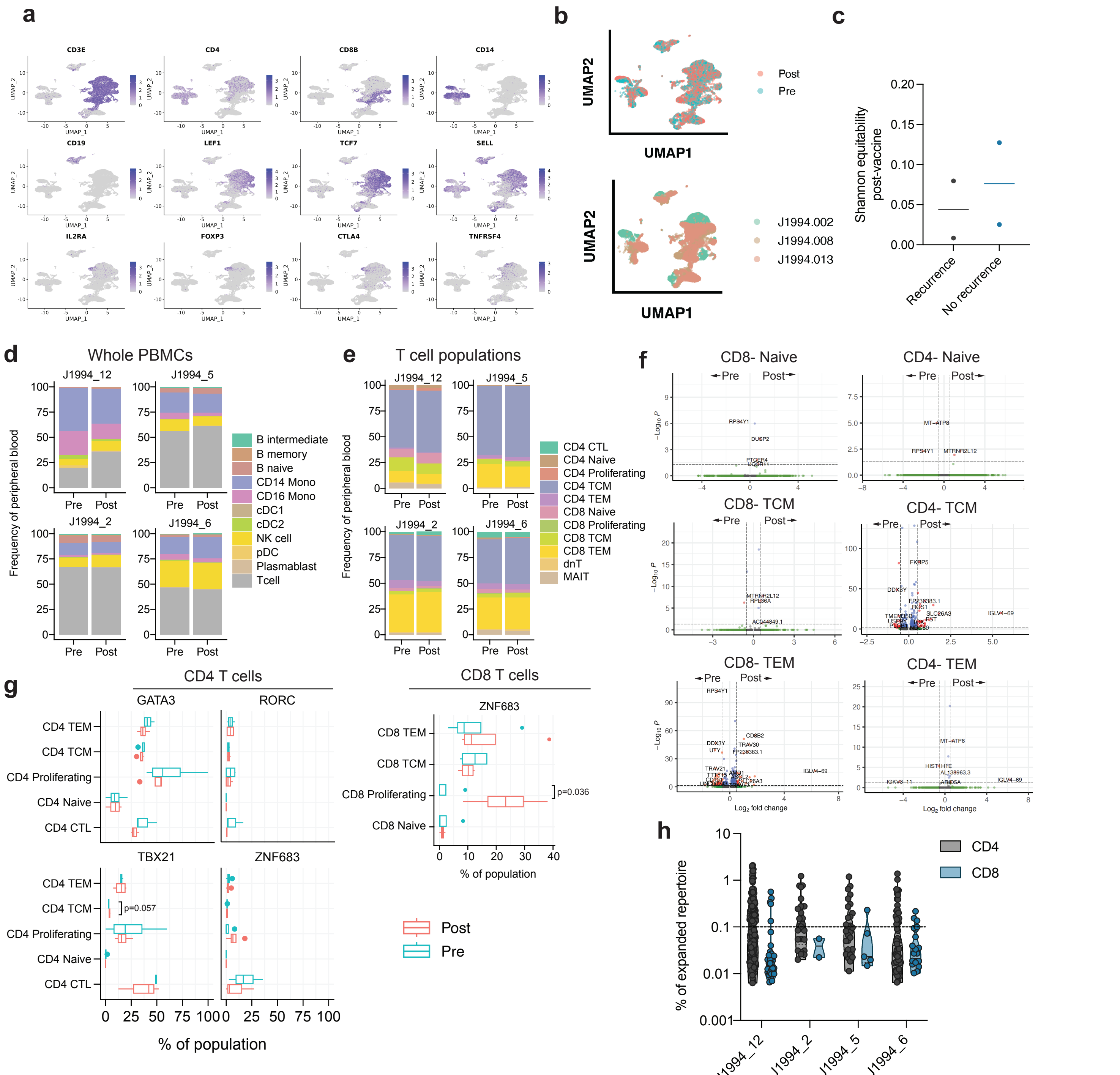

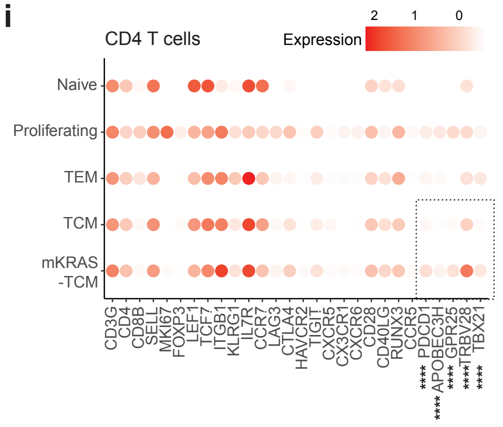

Supplementary Figure 8- Population and gene expression analysis of single cell RNA/TCR sequencing of unstimulated peripheral blood. a) UMAPs of gene marker expression analysis used to identify immune populations in single cell RNA/TCR sequencing of unstimulated peripheral blood. **b)** Overlay of pre- or post- vaccine distribution of cells or patient distribution. **c)** Shannon equitability of the post-vaccine T cell repertoire in patients who recurred compared to no recurrence. **d)** Proportion of all immune cells including B cells (intermediate, memory, plasmablast, or naive), monocytes (CD14 or CD16+), classical (cDC1 or cDC2) or plasmacytoid (pDC) dendritic cells, natural killer (NK) cells, or **e)** T populations including CD4 cytotoxic T lymphocyte (CTL), CD4 and CD8 naïve, CD4 and CD8 proliferating, CD4 and CD8 T central memory (TCM), CD4 and CD8 T effector memory, double negative T cells (dnT), or mucosal associated invariant T cells (MAIT) at pre and post vaccine timepoints. **f)** Differential gene expression analysis T cell populations in CD8 and CD4 naïve or central memory (TCM) or T effector memory (TEM) cells at pre and post vaccine timepoints. **g)** Transcription factor expression at pre- (blue) and post-vaccine (red) timepoints for CD4 (left) or CD8(right). **h)** Frequency of CD4 or CD8 T cells in the mKRAS expanded TCR repertoire that were significantly expanded to mKRAS antigen relative to control for each patient. CD4 or CD8 phenotype was determined by mapping TCRb and V gene onto the single cell RNA/TCRseq of unstimulated peripheral blood. **i)** Gene signature of CD4 T cell compartment of PBMCs including CD4 Naive, CD4 proliferating (Prolif), CD4 T effector memory (TEM), CD4 T central memory (TCM), or mKRAS-specific CD4 TCM cells. Significant-ly upregulated genes are indicated by an asterisk. ns = P 0.05, *P ≤ 0.05, **P ≤ 0.01, ***P ≤ 0.001, ****P ≤ 0.0001. Exact p values for CD4 TCM vs CD4 KRAS TCM are shown in Supp. Table 8.

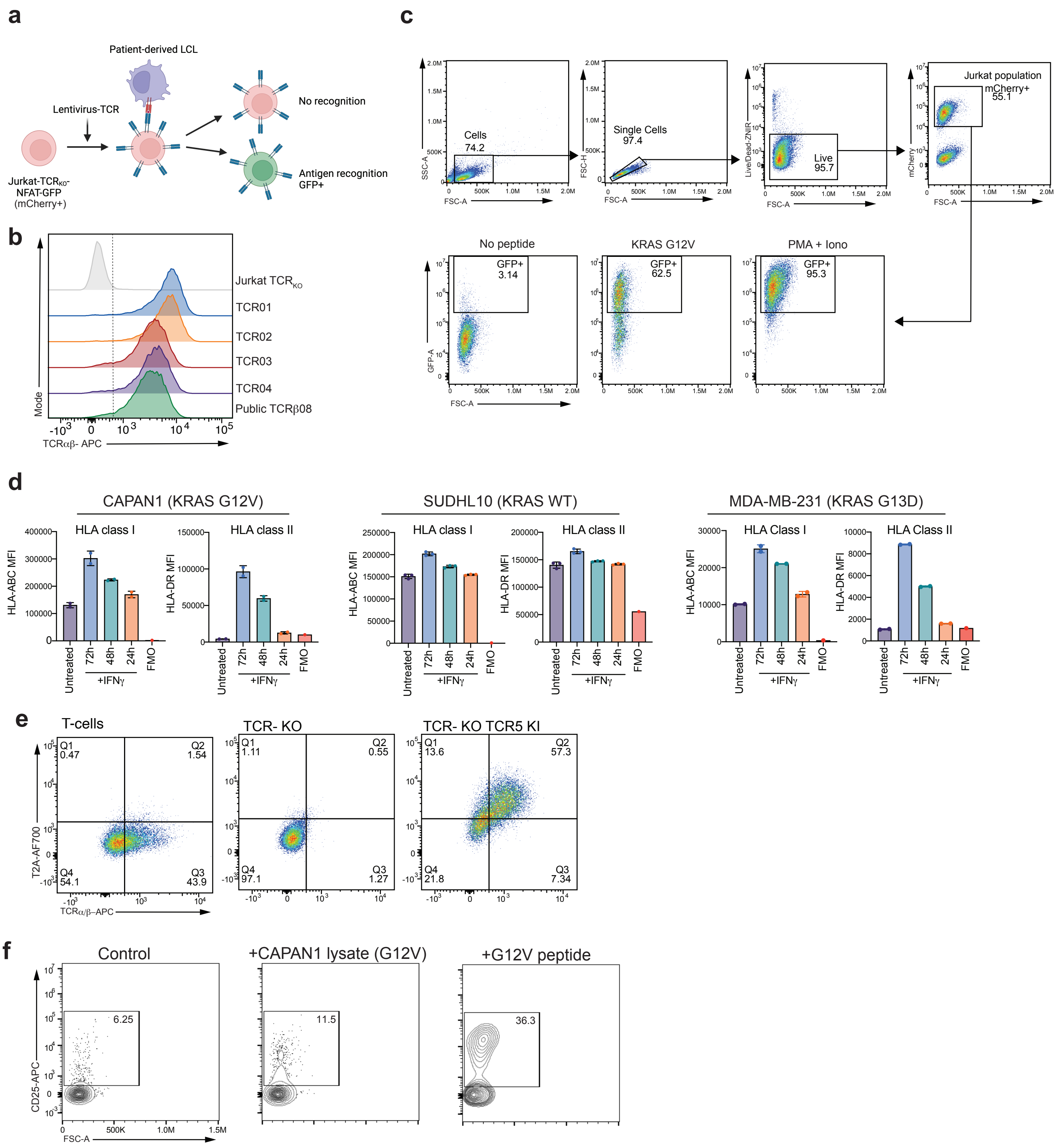

Supplementary Figure 9- Transduction of mKRAS TCRs into Jurkat-TCR$_{KO}$-NFAT-GFP reporter lines and co-culture with peptide pulsed LCLs. **a)** Overview of the workflow for transduction and co-culture of Jurkat-TCR$_{KO}$-NFAT-GFP reporter cells. Jurkat cells consituatively express the mCherry marker. Reporter cells were transduced with lentivirus expressing a TCRα-T2A-TCRβ expression cassette under the control of an EF1α promoter. TCRα/β + flow sorted cells were then co-cultured with peptide-pulsed LCLs at a 1:1 effector to target ratio. TCR activation was measured by GFP expression. Created in Biorender.com, Amanda Huff. (2025) https://BioRender.com/qylx5wh **b)** Validation of TCRα/β expression on TCR transduced jurkats after sorting on TCRα/β+ populations. **c)** Gating scheme for GFP expression analysis on TCR transduced jurkats after co-culture with LCLs pulsed with no peptide, KRAS SLP (G12V shown), or PMA + Ionom cin positive control. Cells were first gated on, then single cells, followed by live cells, and finally mCherry+ (jurkat reporter population). From the mCherry+ population, GFP expression was determined as a percentage. d) Tumor cell lines were treated with 100IU/mL hIFNγ for 24, 48, or 72 hours prior to flow cytometry staining for HLA class I and class II expression relative to an untreated control sample or fluorescent minus one (FMO) control. e) T cells from a healthy donor with isolated and transduced twice with a lentiviral vector expressing mKRAS TCR05 followed by knock out of endogenous TCR using CRISPR/Cas12a tarting the TRAC/TRBC locus. Flow cytometry valdiation of TCR knock out and subsequent TCR05 knock in via flow staining for TCRα/β and T2A-specific antibody detecting cleaved T2A present in the transgenic TCR construct. f) CD25 expression on human T cells expressing TCR05 after co-culture with patient matched LCLs pulsed with control, capan1 tumor lysate (G12V), or G12V peptide.

Supplementary Table 1. Patient demographics and clinical characteristics. Fisher's exact test for categorical and Mann-Whitney test for numerical values was used to determine significance.

**Characteristic**	**All Patients** (*n* = 12)	Recurrence (n = 8)	No Recurrence (n = 4)	P Value*
Age (years)				
Mean (SD)	64.2 (10.5)	64.0 (8.5)	64.5 (15.4)	
Median (range)	68.0 (42.0-76.0)	64.0 (53.0, 76.0)	70.5 (42.0, 75.0)	0.80
Sex				
Male	10 (83.3%)	8 (100%)	2 (50.0%)	0.09
Female	2 (16.7%)	0	2 (50.0%)	
Race				
White	10 (83.3%)	6 (75.0%)	4 (100%)	0.52
Asian	2 (16.7%)	2 (25.0%)	0	
Tumor Location				
Head	7 (58.3%)	4 (50.0%)	3 (75.0%)	0.21
Body	4 (33.3%)	4 (50.0%)	0	
Tail	1 (8.3%)	0	1 (25.0%)	
Tumor Size (cm)				
Mean (SD)	2.78 (0.88)	3.13 (0.82)	2.08 (0.51)	
Median (range)	2.50 (1.50-4.50)	3.25 (2.20, 4.50)	2.10 (1.50, 2.60)	0.09
Tumor Differentiation				
Well-to-moderately differentiated	1 (8.3%)	0	1 (25.0%)	0.24
Moderately differentiated	7 (58.3%)	6 (75.0%)	1 (25.0%)	
Moderate-to-poorly differentiated	2 (16.7%)	1 (12.5%)	1 (25.0%)	
Poorly differentiated	2 (16.7%)	1 (12.5%)	1 (25.0%)	
Pathologic Stage				
IA	1 (8.3%)	0	1 (25.0%)	0.29
IB	2 (16.7%)	1 (12.5%)	1 (25.0%)	
IIB	6 (50.0%)	4 (50.0%)	2 (50.0%)	
III	3 (25.0%)	3 (37.5%)	0	
Lymph Node Status				

N0	3 (25.0%)	1 (12.5%)	2 (50.0%)	0.27
N1	6 (50.0%)	4 (50.0%)	2 (50.0%)	
N2	3 (25.0%)	3 (37.5%)	0	
Resection Margins				
R0	11 (91.7%)	7 (87.5%)	4 (100%)	>0.99
R1	1 (8.3%)	1 (12.5%)	0	
Neoadjuvant Chemotherapy				
(m)FOLFIRINOX	6 (50%)	4 (50.0%)	2 (50.0%)	>0.99
None	6 (50%)	4 (50.0%)	2 (50.0%)	
Adjuvant Chemotherapy				
(m)FOLFIRINOX	9 (75%)	6 (75.0%)	3 (75.0%)	0.75
Gemcitabine/nab-paclitaxel	1 (8.3%)	1 (12.5%)	0	
Gemcitabine/cisplatin/nab-paclitaxel	1 (8.3%)	1 (12.5%)	0	
None	1 (8.3%)	0	1 (25.0%)	
Perioperative Radiation				
Yes	3 (25%)	2 (25.0%)	1 (25%)	>0.99
No	9 (75%)	6 (75.0%)	3 (75%)	
TMB				
Low (<10 mutations per Mbp)	12 (100%)	8 (100%)	4 (100%)	>0.99
High (>10 mutations per Mbp)	0 (0%)	0 (0%)	0 (0%)	
Microsatellite instability (MSI) status				
MSS (microsatellite stable)	11 (91.7%)	8 (100%)	3 (75.0%)	0.33
MSI- high	0 (0%)	0 (0%)	0 (0%)	
MSI- low	0 (0%)	0 (0%)	0 (0%)	
Unknown (not evaluable)	1 (8.3%)	0 (0%)	1 (25.0%)	
Baseline CA-19-9 (U/mL)				
Mean (SD)	23.6 (20.6)	24.4 (23.6)	22.1 (15.9)	
Median (range)	20.1 (0-69.9)	20.1 (0, 69.9)	19.9 (6.40, 42.1)	0.93
KRAS Mutation				
G12D	4 (33.3%)	4 (50.0%)	0	0.27
G12R	2 (16.7%)	1 (12.5%)	1 (25.0%)	
G12V	6 (50%)	3 (37.5%)	3 (75.0%)	

Time From Completion of Adjuvant Chemotherapy to First mKRAS-VAX (months)**	N=11	N=8	N=3	
Mean (SD)	3.9 (1.7)	3.4 (1.7)	5.2 (1.4)	0.08
Median (range)	3.5 (1.6-6.1)	3.0 (1.6-5.5)	5.9 (3.5-6.1)	

**P* values calculated by Fisher's exact test for categorical variables and two-tailed Mann-Whitney test for numerical variables.
**One patient had not received chemotherapy.

Supplementary Table 2. Adverse events related to mKRAS-VAX

**CTCAE Term**	***Grade 1***	***Grade 2***	***Grade 3***	***Grade 4***	***Grade 5***	**Total** (*n* = 12)
Arthralgia	2 (16.7%)	0	0	0	0	2 (16.7%)
Chills	2 (16.7%)	0	0	0	0	2 (16.7%)
Fatigue	4 (33.3%)	0	0	0	0	4 (33.3%)
Fever	4 (33.3%)	0	0	0	0	4 (33.3%)
Headache	1 (8.3%)	0	0	0	0	1 (8.3%)
Malaise	1 (8.3%)	0	0	0	0	1 (8.3%)
Myalgia	2 (16.7%)	0	0	0	0	2 (16.7%)
Nausea	1 (8.3%)	0	0	0	0	1 (8.3%)

**Injection Site Reaction**	***Grade 1***	***Grade 2***	***Grade 3***	***Grade 4***	***Grade 5***	**Total** (*n* = 12)
Erythema	4 (33.3%)	0	0	0	0	4 (33.3%)
Induration	1 (8.3%)	0	0	0	0	1 (8.3%)
Muscle cramp	2 (16.7%)	0	0	0	0	2 (16.7%)
Pain	9 (75.0%)	1 (8.3%)	0	0	0	10 (83.3%)
Pruritus	2 (16.7%)	0	0	0	0	2 (16.7%)
Skin dry	1 (8.3%)	0	0	0	0	1 (8.3%)
Warmth	1 (8.3%)	0	0	0	0	1 (8.3%)

Supplementary Table 3. Adverse events related to ipilimumab

CTCAE Term	*Grade 1*	*Grade 2*	*Grade 3*	*Grade 4*	*Grade 5*	**Total** (*n* = 12)
Adrenal insufficiency	0	1 (8.3%)	1 (8.3%)	0	0	2 (16.7%)
Anorexia	0	1 (8.3%)	0	0	0	1 (8.3%)
Arthralgias	2 (16.7%)	1 (8.3%)	1 (8.3%)	0	0	4 (33.3%)
Arthritis	0	0	0	0	0	0
Chills	1 (8.3%)	0	0	0	0	1 (8.3%)
Colitis	0	1 (8.3%)	0	0	0	1 (8.3%)
Cough	1 (8.3%)	0	0	0	0	1 (8.3%)
Dizziness	1 (8.3%)	0	0	0	0	1 (8.3%)
Dry eye	1 (8.3%)	0	0	0	0	1 (8.3%)
Dry mouth	0	1 (8.3%)	0	0	0	1 (8.3%)
Fatigue	2 (16.7%)	2 (16.7%)	0	0	0	4 (33.3%)
Fever	2 (16.7%)	0	0	0	0	2 (16.7%)
Flatulence	1 (8.3%)	0	0	0	0	1 (8.3%)
Headache	0	1 (8.3%)	0	0	0	1 (8.3%)
Hoarseness	1 (8.3%)	0	0	0	0	1 (8.3%)
Hyperthyroidism	4 (33.3%)	1 (8.3%)	0	0	0	5 (41.7%)
Hypotension	1 (8.3%)	0	0	0	0	1 (8.3%)
Hypothyroidism	2 (16.7%)	3 (25.0%)	0	0	0	5 (41.7%)
Mucositis oral	0	1 (8.3%)	0	0	0	1 (8.3%)
Myalgia	0	0	1 (8.3%)	0	0	1 (8.3%)
Nausea	1 (8.3%)	0	0	0	0	1 (8.3%)
Pneumonitis	0	0	1 (8.3%)	0	0	1 (8.3%)
Pruritus	3 (25.0%)	0	0	0	0	3 (25.0%)
Sinus bradycardia	1 (8.3%)	0	0	0	0	1 (8.3%)
Sinus tachycardia	1 (8.3%)	0	0	0	0	1 (8.3%)
Skin hypopigmentation	0	1 (8.3%)	0	0	0	1 (8.3%)
Thrush	1 (8.3%)	0	0	0	0	1 (8.3%)
Vomiting	1 (8.3%)	0	0	0	0	1 (8.3%)

Supplementary Table 4. Adverse events related to nivolumab

CTCAE Term	*Grade 1*	*Grade 2*	*Grade 3*	*Grade 4*	*Grade 5*	**Total** (*n* = 12)
Adrenal insufficiency	0	1 (8.3%)	1 (8.3%)	0	0	2 (16.7%)
Anorexia	0	1 (8.3%)	0	0	0	1 (8.3%)
Arthralgias	2 (16.7%)	1 (8.3%)	1 (8.3%)	0	0	4 (33.3%)
Arthritis	0	0	0	0	0	0
Chills	1 (8.3%)	0	0	0	0	1 (8.3%)
Colitis	0	1 (8.3%)	0	0	0	1 (8.3%)
Cough	1 (8.3%)	0	0	0	0	1 (8.3%)
Dizziness	1 (8.3%)	0	0	0	0	1 (8.3%)
Dry eye	1 (8.3%)	0	0	0	0	1 (8.3%)
Dry mouth	0	1 (8.3%)	0	0	0	1 (8.3%)
Fatigue	2 (16.7%)	2 (16.7%)	0	0	0	4 (33.3%)
Fever	2 (16.7%)	0	0	0	0	2 (16.7%)
Flatulence	1 (8.3%)	0	0	0	0	1 (8.3%)
Headache	0	1 (8.3%)	0	0	0	1 (8.3%)
Hoarseness	1 (8.3%)	0	0	0	0	1 (8.3%)
Hyperthyroidism	4 (33.3%)	1 (8.3%)	0	0	0	5 (41.7%)
Hypotension	1 (8.3%)	0	0	0	0	1 (8.3%)
Hypothyroidism	2 (16.7%)	3 (25.0%)	0	0	0	5 (41.7%)
Mucositis oral	0	1 (8.3%)	0	0	0	1 (8.3%)
Myalgia	0	0	1 (8.3%)	0	0	1 (8.3%)
Nausea	1 (8.3%)	0	0	0	0	1 (8.3%)
Pneumonitis	0	0	1 (8.3%)	0	0	1 (8.3%)
Pruritus	3 (25.0%)	0	0	0	0	3 (25.0%)
Sinus bradycardia	1 (8.3%)	0	0	0	0	1 (8.3%)
Sinus tachycardia	1 (8.3%)	0	0	0	0	1 (8.3%)
Skin hypopigmentation	0	1 (8.3%)	0	0	0	1 (8.3%)
Thrush	1 (8.3%)	0	0	0	0	1 (8.3%)
Vomiting	1 (8.3%)	0	0	0	0	1 (8.3%)

Supplementary Table 5- Statistics for ELISPOT assays shown in Figure 1C.

Comparison of IFNγ SFU measured for each mKRAS stimulation condition relative to control peptide within 17 weeks of the first mKRAS-VAX. Two way-ANOVA followed by Dunnett's multiple comparisons was performed. Exact p values are shown where significant relative to control. Patient ID and tumor mutation are indicated. Week-post vaccine is approximate.

Week post-vaccine	Comparison vs Control	J1994_1 (G12R)	J1994_2 (G12V)	J1994_3 (G12D)	J1994_5 (G12V)	J1994_6 (G12V)	J1994_7 (G12V)	J1994_9 (G12V)	J1994_10 (G12D)	J1994_12 (G12R)	J1994_13 (G12D)	J1994_14 (G12V)	J1994_18 (G12D)
0	G12V	ns	ns	ns	ns	ns	ns	ns	ns	ns	ns	ns	ns
	G12A	ns	ns	ns	ns	ns	ns	ns	ns	ns	ns	ns	ns
	G12R	ns	ns	ns	ns	ns	ns	ns	ns	ns	ns	ns	ns
	G12C	ns	ns	ns	ns	ns	ns	ns	0.0017	ns	ns	ns	ns
	G12D	ns	ns	ns	ns	ns	ns	ns	ns	ns	ns	ns	ns
	G13D	ns	ns	ns	ns	ns	ns	ns	0.0142	ns	ns	ns	ns
3	G12V	<0.0001	ns	ns	ns	<0.0001	<0.0001	<0.0001	0.0002	<0.0001	0.0005	<0.0001	0.0073
	G12A	<0.0001	ns	0.0001	ns	0.0127	0.0058	<0.0001	ns	<0.0001	0.0002	0.0022	ns
	G12R	<0.0001	ns	ns	0.0014	<0.0001	<0.0001	<0.0001	0.0008	<0.0001	<0.0001	<0.0001	0.0073
	G12C	<0.0001	ns	0.0250	ns	ns	ns	0.0001	<0.0001	<0.0001	0.0002	ns	<0.0001
	G12D	ns	ns	0.0023	ns	ns	ns	0.0235	ns	ns	ns	ns	ns
	G13D	<0.0001	ns	0.0160	ns	<0.0001	ns	0.001	ns	ns	ns	ns	ns
7	G12V	<0.0001	0.0091	<0.0001	<0.0001	<0.0001	<0.0001	0.0059	ns		ns	<0.0001	<0.0001
	G12A	<0.0001	<0.0001	<0.0001	<0.0001	<0.0001	0.0291	ns	ns		ns	ns	ns
	G12R	<0.0001	<0.0001	0.0039	<0.0001	<0.0001	0.0001	<0.0001	ns		ns	ns	<0.0001
	G12C	0.0001	0.0185	<0.0001	<0.0001	0.0027	ns	ns	ns		ns	0.0261	<0.0001
	G12D	ns	<0.0001	<0.0001	ns	ns	ns	ns	ns		ns	ns	0.0076
	G13D	<0.0001	<0.0001	ns	ns	<0.0001	ns	ns	ns		ns	ns	ns
10	G12V	<0.0001	0.0185	ns	<0.0001	<0.0001	<0.0001	ns	<0.0001	<0.0001		0.0022	<0.0001
	G12A	<0.0001	0.0185	0.005	<0.0001	ns	<0.0001	ns	<0.0001	<0.0001		ns	ns
	G12R	<0.0001	<0.0001	ns	<0.0001	<0.0001	<0.0001	<0.0001	0.0008	<0.0001		ns	<0.0001
	G12C	<0.0001	ns	ns	<0.0001	ns	0.0201	ns	<0.0001	<0.0001		ns	<0.0001
	G12D	ns	ns	ns	0.0088	ns	0.0023	ns	ns	0.0012		ns	ns
	G13D	<0.0001	<0.0001	<0.0001	ns	0.0047	ns	ns	ns	0.0296		ns	ns
17	G12V	<0.0001	ns	<0.0001	<0.0001	ns		<0.0001	<0.0001	<0.0001		<0.0001	<0.0001
	G12A	ns	ns	<0.0001	<0.0001	ns		0.0029	0.0002	<0.0001		ns	0.0255
	G12R	<0.0001	<0.0001	0.005	<0.0001	<0.0001		<0.0001	0.0018	<0.0001		ns	<0.0001
	G12C	<0.0001	ns	<0.0001	<0.0001	ns		0.0013	<0.0001	<0.0001		0.0022	<0.0001
	G12D	0.0022	ns	0.0008	ns	0.0079		ns	ns	0.0002		ns	0.0076
	G13D	ns	<0.0001	<0.0001	ns	ns		0.0424	ns	ns		0.0206	ns

Supplementary Table 6- Patient HLA types

Subject	Disease	Mutation	HLA-A	HLA-B	HLA-C	HLA-DRB1	HLA-DQA	HLA-DQB	HLA-DPA	HLA-DPB
J1994_1	PDAC	G12R	01:01	08:01	07:01	07:01	02:01	02:02	01:03	04:01
			25:01	39:01	12:03	11:01	05:05	03:01		
J1994_2	PDAC	G12V	02:01	44:03	04:01	04:02	03:01	03:01	01:03	04:01
			03:01	53:01		11:01	05:05	03:02		
J1994_3	PDAC	G12D	24:02	54:01	1:02	03:01	05:01	02:01	01:03	04:01P
			33:03	58:01	3:02	09:01	03:02	03:03	02:02	05:01P
J1994_5	PDAC	G12V	02:01	13:02	06:02	07:01	02:01	02:02	01:03	04:01P
			30:01	15:01	03:04	15:01	01:02	06:02		04:02P
J1994_6	PDAC	G12V	02:01	27:04	03:04	08:02	04:01	03:01	02:02	05:01
			24:02	40:02	08:01	12:02	06:01	04:02		
J1994_7	PDAC	G12V	03:01	35:08	04:01	07:01		02:02		14:01
			02:01	18:01	07:01	13:01		06:03		
J1994_9	PDAC	G12V	02:01	35:03	04:01	07:01	02:01	03:03	01:03	04:02P
			03:01	57:01	06:02	11:01	05:05	03:01	02:01	13:01P
J1994_10	PDAC	G12D	3:01	7:02	7:02	13:02	1:02	6:04	1:03	1:01
			23:01			15:01		6:02	2:01	4:01
J1994_12	PDAC	G12R	01:01	57:01	06:02	07:01	02:01	03:03	01:03	03:01P
										04:01P
J1994_13	PDAC	G12D	03:01	07:02	07:02	13:01	01:03	06:03	01:03	04:01
			24:02			15:01	01:02	06:02		
J1994_14	PDAC	G12V	01:01	08:01	07:01	03:01	01:02	02:01	01:03	04:01P
				57:01	06:02	13:02	05:01	06:04	02:01	09:01P
J1994_18	PDAC	G12D	02:01	07:02	07:02	01:01	01:01	05:01	01:03	04:01P
			03:01	18:01	07:01	16:01	01:02	05:02	02:01	10:01P

Supplementary Table 7- Statistics for ELISPOT assays shown in Figure 4A.

Comparison relative to control peptide was performed for each mKRAS stimulation condition within the extended treatment phase (>52 weeks post first vaccination). Two way-ANOVA followed by Dunnett's multiple comparisons was performed. Exact p values are shown where significant relative to control. Patient ID and tumor mutation are indicated. Week-post vaccine is approximate.

Week post-vaccine	Comparison vs Control	J1994_5 (G12V)	J1994_1 (G12R)	J1994_2 (G12V)	J1994_14 (G12V)
53	G12V	<0.0001	<0.0001		
	G12A	ns	ns		
	G12R	ns	<0.0001		
	G12C	0.0217	ns		
	G12D	ns	ns		
	G13D	ns	0.0010		
65	G12V	ns	<0.0001	<0.0001	<0.0001
	G12A	ns		ns	ns
	G12R	ns	<0.0001	ns	0.0122
	G12C	ns	<0.0001	0.0289	ns
	G12D	ns		ns	ns
	G13D	ns	0.0009	ns	ns
80	G12V	<0.0001	<0.0001	ns	<0.0001
	G12A	<0.0001	<0.0001	ns	ns
	G12R	0.0023	<0.0001	<0.0001	ns
	G12C	<0.0001	0.0049	0.0047	ns
	G12D	ns	0.0022	ns	ns
	G13D	ns	0.0110	0.0002	ns
95	G12V	<0.0001			
	G12A	0.0002			
	G12R	ns			
	G12C	0.0073			
	G12D	ns			
	G13D	ns			
105	G12V	ns			
	G12A	ns			
	G12R	ns			
	G12C	ns			
	G12D	ns			
	G13D	ns			

Supplementary Table 8- Differential gene expression between mKRAS-specific CD4 Tcm cells and all other CD4 Tcm cells in the single cell RNA/TCRseq data set. A Wilcoxon Rank Sum test was performed for significance.

	p value	avg_log2FC	CD4 Tcm KRAS	CD4 Tcm	adjusted p value
PDCD1	1.13E-19	2.14723	0.248	0.052	3.79E-15
APOBEC3H	7.33E-16	2.554234	0.128	0.019	2.46E-11
GPR25	3.98E-13	2.16401	0.174	0.039	1.33E-08
TRBV28	4.71E-11	2.58221	0.248	0.081	1.58E-06
TBX21	8.70E-09	2.394041	0.138	0.035	0.000291806

Supplementary Table 9- Peptides that were excluded at each vaccination cycle for all patients.
If peptide stability or sterility dropped below acceptable standards, listed peptides were excluded from the pooled vaccine at the indicated cycle timepoints. C indicates cycle of treatment, D indicates day of cycle. Patient ID and tumor-specific mutation are indicated.

	C1D1	C1D8	C1D15	C2	C3	C4	C5	C6	C7	C8	C9	C10	C11	C12	C13	C14	C15	C16	C17	C18
**J1994_1**<br>G12R					G12C,<br>G12A												G12A	G12A	G12A	G12A
**J1994_2**<br>G12V	G12C,<br>G12A	G12C,<br>G12A	G12C,<br>G12A	G12C,<br>G12A													G12A	G12A	G12A	G12A
**J1994_3**<br>G12D																				
**J1994_5**<br>G12V															G12C,<br>G12A					G12A
**J1994_6**<br>G12V	G12C,<br>G12A	G12C,<br>G12A	G12C,<br>G12A	G12C,<br>G12A																
**J1994_7**<br>G12V	G12C,<br>G12A	G12C,<br>G12A	G12C,<br>G12A	G12C,<br>G12A			G12C<br>G12A													
**J1994_9**<br>G12V							G12C,<br>G12A													
**J1994_10**<br>G12D							G12C,<br>G12A													
**J1994_12**<br>G12R																				
**J1994_13**<br>G12D																				
**J1994_14**<br>G12V															G12A		G12A	G12A	G12A	G12A
**J1994_18**<br>G12D	G12A	G12A	G12A	G12A			G12A		G12A											

Supplementary Table 10- Panel of antibodies used for CyTOF profiling of peptide restimulated mKRAS-specific T cells

Mass	Metal	Antigen	Clone	Dilution Factor	Source	Cocktail
89	Yt	CD45	HI30	200	Standard BioTools™	Barcode
103	Rh	Cell ID - Intercalator	N/A	500	Standard BioTools™	Cell ID
110-116	Cd	CD45	HI30	200	Standard BioTools™	Barcode
141	Pr	CD196/CCR6	G034E3	120	Standard BioTools™	Chemokine
142	Nd	CD19	HIB19	100	Standard BioTools™	Surface
143	Nd	HLA-DR	L243	200	Standard BioTools™	Surface
144	Nd	CD195/CCR5	NP-6G4	120	Standard BioTools™	Chemokine
145	Nd	CD4	RPA-T4	200	Standard BioTools™	Surface
146	Nd	CD8	RPA-T8	200	Standard BioTools™	Surface
147	Sm	CD7	CD7-6B7	200	Standard BioTools™	Surface
148	Nd	CD14	RMO52	100	Standard BioTools™	Surface
149	Sm	CD25	2A3	400	Standard BioTools™	Surface
150	Nd	CD134/OX40	ACT35	200	Standard BioTools™	Surface
151	Eu	CD2	TS1/8	400	Standard BioTools™	Surface
152	Sm	TNFa	Mab11	133	Standard BioTools™	Intracellular
153	Eu	CD366/Tim-3	F38-2E2	100	Standard BioTools™	Surface
154	Sm	TIGIT	MBSA43	100	Standard BioTools™	Surface
155	Gd	CD56	B159	400	Standard BioTools™	Surface
156	Gd	CD274/PDL-1	29E.2A3	100	Standard BioTools™	Surface
158	Gd	IL-2	MQ1-17H12	133	Standard BioTools™	Intracellular
159	Tb	CD197/CCR7	G043H7	120	Standard BioTools™	Chemokine
160	Gd	CD28	CD28.2	200	Standard BioTools™	Surface
161	Dy	CD152/CTLA-4	14D3	200/264	Standard BioTools™	Surface/Intracellular
162	Dy	FoxP3	PCH101	133	Standard BioTools™	Intracellular
163	Dy	CD183/CXCR3	G025H7	120	Standard BioTools™	Chemokine
165	Ho	CD45RO	UCHL1	100	Standard BioTools™	Surface
166	Er	CD314/NKG2D	ON72	200	Standard BioTools™	Surface
167	Er	CD27	O323	200	Standard BioTools™	Surface
168	Er	IFNg	B27	66	Standard BioTools™	Intracellular
169	Tm	CD45RA	HI100	400	Standard BioTools™	Surface
170	Er	CD3	UCHT1	200	Standard BioTools™	Surface

171	Yb	Granzyme B	GB11	66	Standard BioTools™	Intracellular
172	Yb	Ki-67	B56	66	Standard BioTools™	Intracellular
173	Yb	CD137/4-1BB	4B4-1	200	Standard BioTools™	Surface
174	Yb	CD279/PD-1	EH12.2H7	100	Standard BioTools™	Surface
175	Lu	CD223/LAG-3	11C3C65	100	Standard BioTools™	Surface
176	Yb	CD127/IL-7Ra	A019D5	100	Standard BioTools™	Surface
194-198	Pt	Cell ID - Cisplatin	N/A	1000	Standard BioTools™	Cell ID
209	Bi	CD16	3G8	100	Standard BioTools™	Surface
