# Supplementary material for: Mutant KRAS vaccine with dual checkpoint blockade in resected pancreatic cancer: a phase I trial
Source: Nat Commun. 2026 Feb 10;17:1538. doi: 10.1038/s41467-026-68324-4 (PMC12891733; doi:10.1038/s41467-026-68324-4)
Supplement: Supplementary file 2 — Reporting Summary [file 41467_2026_68324_MOESM2_ESM.pdf]

nature portfolio

Corresponding author(s): Neeha Zaidi

Last updated by author(s): Aug 19, 2025

# Reporting Summary

Nature Portfolio wishes to improve the reproducibility of the work that we publish. This form provides structure for consistency and transparency in reporting. For further information on Nature Portfolio policies, see our Editorial Policies and the Editorial Policy Checklist.

## Statistics

For all statistical analyses, confirm that the following items are present in the figure legend, table legend, main text, or Methods section.

| n/a | Confirmed | |
|---|---|---|
| ☐ | ☒ | The exact sample size (*n*) for each experimental group/condition, given as a discrete number and unit of measurement |
| ☐ | ☒ | A statement on whether measurements were taken from distinct samples or whether the same sample was measured repeatedly |
| ☐ | ☒ | The statistical test(s) used AND whether they are one- or two-sided<br>*Only common tests should be described solely by name; describe more complex techniques in the Methods section.* |
| ☐ | ☒ | A description of all covariates tested |
| ☐ | ☒ | A description of any assumptions or corrections, such as tests of normality and adjustment for multiple comparisons |
| ☐ | ☒ | A full description of the statistical parameters including central tendency (e.g. means) or other basic estimates (e.g. regression coefficient) AND variation (e.g. standard deviation) or associated estimates of uncertainty (e.g. confidence intervals) |
| ☐ | ☒ | For null hypothesis testing, the test statistic (e.g. *F*, *t*, *r*) with confidence intervals, effect sizes, degrees of freedom and *P* value noted<br>*Give P values as exact values whenever suitable.* |
| ☒ | ☐ | For Bayesian analysis, information on the choice of priors and Markov chain Monte Carlo settings |
| ☒ | ☐ | For hierarchical and complex designs, identification of the appropriate level for tests and full reporting of outcomes |
| ☐ | ☒ | Estimates of effect sizes (e.g. Cohen's *d*, Pearson's *r*), indicating how they were calculated |

*Our web collection on statistics for biologists contains articles on many of the points above.*

## Software and code

Policy information about availability of computer code

| | |
|---|---|
| Data collection | *Provide a description of all commercial, open source and custom code used to collect the data in this study, specifying the version used OR state that no software was used.* |
| Data analysis | Single cell sequencing data was processed using the Cellranger 5.0.1 pipeline (10x Genomics). Raw feature-barcode matrices were imported into Seurat version 4.3 for processing56. Cells were clustered following QC using the Seurat FindClusters function using resolution 0.3. T cell clusters were differentiated from other PBMCs by expression patterns of positive and negative markers including CD3E, CD4, CD8B, CD14, CD19, FCGR3A, the presence of VDJ reads, and predicted cell type using Azimuth version 0.5.0 (PBMC reference, level 2 resolution). Gene expression analyses performed using native Seurat functions and custom analysis in R version 4.3.1. T-cell receptor sequencing data were analyzed in R to identify significantly expanded, antigen-specific clonotypes using a fisher's exact test. All relevant code is available at http:/ doi.org/10.5281/zenodo.11186766.<br><br>For CyTOF analysis, Acquired data was preprocessed for normalization, randomization, and bead removal using CyTOF Software (v6.7, Standard BioTools). Gating for cell events, live cells, and debarcoding was performed using FlowJo (v10.9, BD). Processed FCS files were loaded into R (v4.0.2) for analysis. Cells were clustered using FlowSOM algorithm. |

For manuscripts utilizing custom algorithms or software that are central to the research but not yet described in published literature, software must be made available to editors and reviewers. We strongly encourage code deposition in a community repository (e.g. GitHub). See the Nature Portfolio guidelines for submitting code & software for further information.

## Data

Policy information about availability of data

All manuscripts must include a data availability statement. This statement should provide the following information, where applicable:

- Accession codes, unique identifiers, or web links for publicly available datasets
- A description of any restrictions on data availability
- For clinical datasets or third party data, please ensure that the statement adheres to our policy

Upon publication, datasets and corresponding accession numbers will be made available in the manuscript for at the following repositories: Single cell RNA/TCR sequencing data at dbGAP for source files  and GEO for analysis files, bulk TCRseq files is available at GEO, CyTOF data is available at Zenodo.

## Research involving human participants, their data, or biological material

Policy information about studies with human participants or human data. See also policy information about sex, gender (identity/presentation), and sexual orientation and race, ethnicity and racism.

| | |
|---|---|
| Reporting on sex and gender | Both male and female participants were included in this study. Sex and gender were recorded based on clinical documentation in the electronic medical record based on self-report. Analyses were not stratified by sex/gender because the study was not powered to detect sex-specific differences in outcomes. |
| Reporting on race, ethnicity, or other socially relevant groupings | Self-reported race and ethnicity were collected from the electronic medical record as part of standard clinical documentation. Race/ethnicity was not used as an inclusion or exclusion criterion. Analyses were not stratified by race/ethnicity, as the study was not powered to detect differences across these subgroups. |
| Population characteristics | The study population consisted of 12 adult patients with resected pancreatic cancer who were treated at Johns Hopkins. All patients had tumors harboring one of six KRAS mutations included in the study vaccine (KRAS G12D, G12V, G12R, G12C, G12A, G13D). |
| Recruitment | Participants were recruited from Johns Hopkins under an IRB-approved protocol. Patients were approached at the time of clinic visits and invited to participate if they met eligibility criteria. No financial incentives were provided. Recruitment was not restricted by sex, race, or ethnicity, and all eligible patients were offered participation. Informed consent was obtained from all participants prior to enrollment. |
| Ethics oversight | This trial was approved by the Johns Hopkins IRB. |

Note that full information on the approval of the study protocol must also be provided in the manuscript.

# Field-specific reporting

Please select the one below that is the best fit for your research. If you are not sure, read the appropriate sections before making your selection.

☒ Life sciences ☐ Behavioural & social sciences ☐ Ecological, evolutionary & environmental sciences

For a reference copy of the document with all sections, see nature.com/documents/nr-reporting-summary-flat.pdf

# Life sciences study design

All studies must disclose on these points even when the disclosure is negative.

| | |
|---|---|
| Sample size | 12 patients were enrolled and treated in this Phase I study designed to test safety and immunogenicity as the co-primary endpoints. |
| Data exclusions | No data was excluded from this analysis. |
| Replication | The data reported here were reproducible. |
| Randomization | This was a single arm study and therefore randomization was not applicable. |
| Blinding | This was an open-label phase I study and therefore blinding was not applicable. |

# Reporting for specific materials, systems and methods

We require information from authors about some types of materials, experimental systems and methods used in many studies. Here, indicate whether each material, system or method listed is relevant to your study. If you are not sure if a list item applies to your research, read the appropriate section before selecting a response.

## Materials & experimental systems

| n/a | Involved in the study | |
|---|---|---|
| | x | Antibodies |
| | x | Eukaryotic cell lines |
| x | | Palaeontology and archaeology |
| x | | Animals and other organisms |
| | x | Clinical data |
| x | | Dual use research of concern |
| x | | Plants |

## Methods

| n/a | Involved in the study | |
|---|---|---|
| x | | ChIP-seq |
| | x | Flow cytometry |
| x | | MRI-based neuroimaging |

# Antibodies

Antibodies used

For flow cytometry analysis the following antibodies were used: anti-CD3-FITC (clone HIT3a Biolegend cat #300306) , anti-CD4-BV605 (clone RPA-T4, Biolegend cat#300556), anti-CD8 -BV421 (clone RPA-T8, Biolegend cat#301036), anti-CD45RO-AF700 (clone UCHL1, cat#304218), anti-CD62L-PerCPCy5.5 (clone DREG-56, Biolegend cat# 304823), anti-CD69-PE (clone FN50, Biolegend cat#310905), anti-CD137-PECy7 (clone 4B4-1, Biolegend cat#309817) in FACS buffer. Samples were washed twice with FACS buffer and then permeabilized with BD Perm/Fix kit (BD Biosciences, cat# 554714) followed by staining with anti-IFNγ-APC (clone 4S.B3, Biolegend cat#502512), anti-IL-2-PE/Dazzle (clone MQ1-17H12, Biolegend cat#500343), anti-TNFα-BV650 (clone Mab11, Biolegend cat#502938).

For CyTOF analysis, the antibodies used along with clone, metal conjugate, and company origin are found in Supplemental Table 10.

Validation

Each antibody used was validated by the manufacturer.

# Eukaryotic cell lines

Policy information about cell lines and Sex and Gender in Research

Cell line source(s)

Fresh human PBMCs were isolated from leukophoresis byproducts collected at the Johns Hopkins blood donation center. Sex was not reported to the researchers at time of delivery. Jurkat cell lines pASP90 and pASP90-CD8 were a kind gift from Dr. Beatriz Careno. SUDHL10, K562, CAPAN1, MDA-MB-231 were obtained from ATCC.

Authentication

Cell lines were authenticated per ATCC.

Mycoplasma contamination

Cell lines were tested for Mycoplasma contamination at the Johns Hopkins Genetic Resources Core Facility.

Commonly misidentified lines
(See ICLAC register)

*Name any commonly misidentified cell lines used in the study and provide a rationale for their use.*

# Clinical data

Policy information about clinical studies

All manuscripts should comply with the ICMJE guidelines for publication of clinical research and a completed CONSORT checklist must be included with all submissions.

Clinical trial registration

NCT04117087

Study protocol

The study protocol is provided in the supplementary files.

Data collection

Clinical data were collected prospectively at the time of enrollment and during protocol-defined visits. Demographic variables, disease characteristics, and treatment information were abstracted from the electronic medical record. Radiographic assessments were performed at prespecified intervals. Laboratory and correlative studies were performed on prospectively collected biospecimens. All data were recorded in a secure, IRB-approved research database. All participants provided written informed consents.

Outcomes

The co-primary endpoints were safety and immunogenicity. Safety was defined by the incidence and severity of treatment-related adverse events, graded according to the NCI Common Terminology Criteria for Adverse Events (CTCAE), version 5.0. Immunogenicity was defined by the maximal fold change in mutant KRAS-specific T cells within 17 weeks post-vaccination, as measured by IFN-gamma ELISPOT.

## Plants

Seed stocks: N/a

Novel plant genotypes: N/a

Authentication: N/a

# Flow Cytometry

## Plots

Confirm that:

- [x] The axis labels state the marker and fluorochrome used (e.g. CD4-FITC).
- [x] The axis scales are clearly visible. Include numbers along axes only for bottom left plot of group (a 'group' is an analysis of identical markers).
- [x] All plots are contour plots with outliers or pseudocolor plots.
- [x] A numerical value for number of cells or percentage (with statistics) is provided.

## Methodology

Sample preparation: For PBMC staining, frozen PBMCs were thawed in a 37C water bath, plated, and rested overnight before stimulation. Stimulated T cells were treated with golgi stop 5 hours prior to collection and staining. For target cell- T cell coculture systems, co-cultured cells were collected and stained after the time indicated in the methods section. For staining, cells were washed 2X with PBS and then stained with Zombie NIR for 15 minutes at room temperature. Cells were then washed 2X with cold FACS buffer and then stained with extracellular antibody cocktail for 20 minutes at 4C. Cells were then washed 2X with FACS buffer. If intracellular staining was performed, cells were permeabilized in BD Fix/Perm buffer for 20 minutes 4C, washed 2X with 1X Perm/wash buffer, and stained with intracellular antibody cocktail for 30 minutes at 4C. Cells were then washed 2X with 1X Perm/wash and resuspended in FACS buffer to run.

Instrument: Beckman Coulter CytoFLEX

Software: CytExpert was used for collection. FlowJo version 10.8.1 was used for analysis.

Cell population abundance: All cell abundance populations are indicated in reported flow plots. TCR transduced jurkats were sorted and confirmed to be >95% TCR+ prior to use.

Gating strategy: Cells were first gated on a general cell population by FSC-A and SSC-A. Single cells were isolated by gating on FSC-H by FSC-A. Live cells were then gated on in the negative staining fraction of the Zombie NIR.

- [x] Tick this box to confirm that a figure exemplifying the gating strategy is provided in the Supplementary Information.
